# Systematic Screening Study for the Selection of Proper Stabilizers to Produce Physically Stable Canagliflozin Nanosuspension by Wet Milling Method

**DOI:** 10.3390/bioengineering10080927

**Published:** 2023-08-04

**Authors:** Yagmur Pirincci Tok, Burcu Mesut, Sevgi Güngör, Ali Osman Sarıkaya, Emre Erol Aldeniz, Udaya Dude, Yıldız Özsoy

**Affiliations:** 1 Institute of Health Sciences, Istanbul University, Istanbul 34216, Türkiye; yagmur.pirincci@ogr.iu.edu.tr; 2 Department of Pharmaceutical Technology, Faculty of Pharmacy, Istanbul University, Istanbul 34126, Türkiye; bmesut@istanbul.edu.tr (B.M.); sgungor@istanbul.edu.tr (S.G.); 3 Research and Development Center, Abdi İbrahim Pharmaceutical Company, İstanbul 34538, Türkiye; ali.sarikaya@abdiibrahim.com.tr (A.O.S.); emre.aldeniz@abdiibrahim.com.tr (E.E.A.); udaya.dude@abdiibrahim.com.tr (U.D.)

**Keywords:** nanocrystal technology, nanosuspension, wet-milling, quality by design, physical stability

## Abstract

One of the crucial approaches to managing the low solubility and weak bioavailability of drugs is via nanocrystal technology. Through this technology, drug particles have an increased solubility and a faster dissolution rate due to high surface free energy, which requires an appropriate stabilizer(s) to prevent instabilities during the manufacturing process and storage of the nanosuspension. This study aimed to establish a scientific predictive system for properly selecting stabilizers or to reduce the attempts on a trial-and-error basis in the wet-milling method. In total, 42 experiments were performed to examine the effect of critical material attributes on the wettability of the drug, the saturation solubility in the stabilizer solutions or combinations thereof and the dynamic viscosity of stabilizer solutions. All data were evaluated by Minitab 19^®^ and an optimization study was performed. The optimized formulation at a certain concentration of stabilizer combination was ground by Dyno Mill^®^ with 0.3 mm beads for one hour. The optimized nanosuspension with a particle size of 204.5 nm was obtained in short milling time and offered 3.05- and 3.51 times better dissolution rates than the marketed drug product (Invokana^®^ 100 mg) in pH 4.5 and pH 6.8 as non-sink conditions, respectively. The formulation was monitored for three months at room temperature and 4 °C. The parameters were 261.30 nm, 0.163, −14.1 mV and 261.50 nm, 0.216 and −17.8 mV, respectively. It was concluded that this approach might indicate the appropriate selection of stabilizers for the wet-milling process.

## 1. Introduction

A significant portion of drugs in the pharmaceutical market are known to suffer from low oral bioavailability, while 70–90% of new chemical entities (NCEs) are estimated to have poor water solubility [1,2]. The restricted dissolution rate due to poor solubility often leads to a low bioavailability of orally administered drugs, which have a solubility of less than 100 µg/mL [3,4].

Various techniques, including solid dispersion, emulsion, liposomes, complexation, salt formation and cyclodextrin inclusion, have been made to develop the dissolution and solubility of these drugs. However, nanocrystals have become more desirable than other techniques due to high drug loading, reduced toxicity and positive effects on pharmacokinetics, drug absorption and biodistribution [5,6]. Nanocrystals are drug particles in the nanometer range and are called nanosuspension when produced in a dispersion medium. Decreasing the particle size to this level leads to an increase in surface area and subsequently provides an enhanced dissolution rate as explained by the Noyes–Whitney equation and an enhanced solubility, which is caused by a better curvature of the drug particles [7,8].

The only limitation of nanosuspension is the issue of physical stability, i.e., a thermodynamically unstable system. Essential advantages may be lost if the agglomeration or ripening of nanocrystals occur [9]. In particular, ground submicron particles produced by wet stirred media mills (WSMMs) tend to frequently agglomerate due to a high solid concentration, an improved Brownian motion, little distance between particles, and a higher Gibbs free energy. The high-speed rotation of the agitator shaft causes a turbulent shear and translation motion followed by high collision frequencies between the particle–particle, the particle and the grinding media, which lastly result in agglomeration frequencies [10,11]. Therefore, stabilizer agents are necessary for nanosuspension, and the selection of appropriate stabilizers is crucial both during the wet-milling of nanosuspensions and their storage [12]. However, the selection of stabilizer agents and the determination of their concentration are difficult and important tasks, since otherwise, they would excessively raise the solubility of nanosuspension and may raise Ostwald ripening [13]. As mentioned in the Kelvin equation, small particles show a more advanced saturation solubility than larger ones, and this situation resulted in a concentration gradient between them. Particles transfer from small particles with a higher concentration to larger ones with a lower drug concentration, which causes Ostwald ripening.

There are many studies in the literature on the selection of suitable stabilizers. Initial studies have provided an improved understanding of the overall relationship between drugs and polymers, but were also based on a trial-and-error approach [14,15]. Some studies preferred to focus on drug- and time-sparing approaches rather than predictive ones, but such approaches need more specific techniques like dynamic equilibrium curves and adsorption isotherms [10,16]. Recent studies have tried to identify suitable stabilizers by more practical and easier techniques, including determining drug wettability alone or measuring milled particle size after selecting some excipients according to the results of the saturation solubility of the drug in the stabilizer or their combination solution. But most of these studies still have a trial-and-error approach and show a lack of systematic screening [17,18].

The quality by design concept presents a more scientific, efficient approach to establish quality rather than testing the product. Experimental design is a useful tool to evaluate interaction between independent factors and outputs in the scope of the QbD [19,20].

Canagliflozin (CFZ), as the first member of the SGLT2 inhibitor, lowers blood glucose degree through an insulin-independent mechanism. In other words, it increases urinary glucose excretion in patients with type 2 diabetes mellitus (T2DM) and also positively affects weight loss and blood pressure lowering [21]. Canagliflozin is rapidly absorbed after 100 or 300 mg of oral administration and reaches peak plasma concentration within 1–2 h [22]. Nearly 60% and 33% of the dose was excreted in feces and urine, respectively [23]. It is largely metabolized via O-glucuronidation and thus has an oral bioavailability of 65% [22]. The drug is classified as BCS Class IV but has intermediate permeability [24,25]. Hence, this drug is considered a suitable candidate for applying nanocrystal technology.

In this study, we aimed a systematic and predictive screening study to select the proper stabilizer agent(s) using the quality by design (QbD) roadmap for CFZ nanosuspension produced by a large-capacity WSMM. Rather than screening stabilizer agents with small-scale equipment, it is a more difficult and material-consuming process with WSMMs. Thus, this alternative approach emerged when small-scale equipment was not available in the industry. To the authors’ best knowledge, the QbD approach was used for the first time to predict stabilizer type and concentration. After obtaining one stable nanosuspension with one polymer (HPMC-E15) and surfactant (SLS), further attempts have not been made with WSMMs for screening other stabilizers. The wettability of the drug, the saturation solubility in the stabilizer solutions or combinations thereof and the dynamic viscosity of stabilizer solutions were evaluated by Minitab 19^®^, and an optimization study was performed to determine the final stabilizers agent(s) and their concentration.

## 2. Materials and Methods

### 2.1. Materials

Canagliflozin (hemihydrate, D_90_: 18.119 µm) was purchased from Fuxin Long Rui Pharmaceutical CO., Ltd. (Fuxin, China). Hydroxyl propyl methyl cellulose 15 cP (Methocel^®^E15 LV) was obtained from Colorcon Middle East Pvt. Lt. (Istanbul, Turkey). Soluplus^®^, poloxamer 188 (P188, Kolliphor^®^ P188), poloxamer 407 (P407, Kolliphor^®^ P407), sodium lauryl sulfate (SLS, Kolliphor^®^ SLS fine), polyvinylpyrrolidone—polyvinyl acetate copolymers (PVP VA64, Kollidon^®^ VA 64) and polyvinylpyrrolidone (PVP K30, Kollidon^®^ 30) were obtained from BASF, Ludwigshafen, Germany. Polysorbate 80 (Tween^®^ 80) and polysorbate 20 (Tween^®^ 20) were taken from Sigma-Aldrich Chemie GmbH, Steinheim, Germany. Methanol, acetonitrile (HPLC grade) and 85% ortho-phosphoric acid were obtained from MERCK Millipore, Darmstadt, Germany and ISOLAB, Wertheim, Germany, respectively. Ultrapure water was prepared by using a Sartorius Arium Pro purification system.

### 2.2. Preformulation Studies with WSMM

Wet stirred media milling of CFZ was conducted using DYNO^®^-MILL (model: ECM-AP 05) (WAB, Muttenz, Switzerland). The coolable grinding chamber covered with a grinding cylinder made of silicon carbide has a volume of 0.5 L and 60–70% of the volume must be filled with grinding beads for an efficient grinding process. The suspension to grind is pumped from a feed tank into the grinding chamber. Inside the chamber, ceramic accelerators form mixing circuits of grinding beads and the initial suspension. At the grinding chamber outlet, the grinding beads are separated from the suspension by the WAB separation system and then returned to the accelerators. The suspension is then re-pumped from the feed tank into the grinding chamber in circulation mode. The heat is kept under control by means of central cooling that circuits inside the cooling cylinder of the grinding chamber. In all experiments, the filling volume was 65% of the chamber with 0.3 mm grinding beads made from yttrium-stabilized zirconium oxide; the agitator speed was 2998 1/min (equivalent to 10 m/s peripheral speed); the pump speed was 40 rpm.

All percentages (%) are expressed as *w*/*w* with regard to the total weight of suspension, which refers to coarse drug particles in the stabilizer solution. In light of the literature, HPMC-E15 and SDS were selected as the most used stabilizers for initial studies. The CFZ suspension was prepared by dissolving HPMC and SDS, followed by dispersing 5% coarse CFZ drug particles at room temperature for 30 min, and mixing was carried out by a shear mixer at 450 rpm (Fisher Scientific, Pittsburgh, PA, USA). After 30 min, the particle size was measured as the initial value before grinding. The CFZ nanosuspension was milled for one hour at 20–22 °C. Also, a small amount of aliquots were taken at specific time points during milling and then freshly measured to monitor the pattern of particle size reduction. The wet-milled suspension was stored for one month at room temperature (22 °C ± 2 °C) and in the refrigerator (4 °C ± 2 °C). Mean particle size and zeta potential were measured to evaluate physical stability.

#### 2.2.1. Characterization of Nanosuspension during Formulation Development

##### Particle Size and Zeta Potential Measurement

The mean particle size of the suspension to grind was measured by laser diffraction (LD) technique using Mastersizer 2000 equipped with Hydro 2000S (Malvern Instruments, Malvern, UK). The refractive index of CFZ and dispersion media (as water) were 1.324 and 1.330, respectively. The average particle size, polydispersity index and zeta potential of CFZ nanosuspension were measured by dynamic light scattering (DLS) using Litesizer 500 (Anton Paar, Graz, Austria). Before measurement, the nanosuspension was diluted 100 times with deionized water. All measurements were performed 3 times at room temperature.

##### Determination of Drug Content

The assay method was modified by Kaur et al. and validated according to ICH Q2 [26]. The analysis was performed on a Waters Alliance HPLC 2695 separations module connected to a Waters 2996 Photodiode Array Detector set at 290 nm. Data acquisition was performed using Empower^®^ 3 software. Chromatographic analysis was carried out using a C18 ACE column (250 mm × 4.5 mm, 5 mm). The mobile phase comprises 0.1% *v*/*v* ortho-phosphoric acid and acetonitrile in the ratio of 45:55 (*v*/*v*). The mobile phase was degassed for 20 min by sonication using an ultrasonic bath (Elmasonic S 180, Singen, Germany). The injection volume was 10 µL with a flow rate of 1 mL/min; the column oven temperature was 30 °C ± 2 °C and the sample temperature of 25 °C ± 2 °C.

Nanosuspension equivalent to 10 mg CFZ was dissolved with mobile phase in a volumetric flask and samples were prepared at 100% concentration (16.7 µg/mL), sonicated and then filtered with 0.20 µm RC filter (Sartorius, AG, Goettingen, Germany). The analysis was performed in triplicate. The drug content was calculated using the following equation:

Drug content (%) = (Observed drug content/Theoretical drug content) × 100.

### 2.3. Selection of Final Stabilizers by Statistical Design of Experiment (DoE)

After obtaining a single stable nanosuspension formulation with the combination of HPMC- E15 and SDS solution, these parameters, which we observed from laboratory experience and are supported by the literature, were identified as critical quality attributes (CQAs) related to stabilizers. With this combination of stabilizer solution, the wettability of the drug, saturation solubility of the drug in the solution and dynamic viscosity of the solution were examined.

It should be noted that producing a nanosuspension with a particle size below 400 nm and with physical stability is a multifactorial phenomenon such as drug concentration, agitator shaft speed, bead size, filling volume, flow rate, milling time and temperature. The mentioned parameters were kept constant in this study to evaluate the only effect of stabilizers on obtaining a physically stable nanosuspension with the smallest particle size.

HPMC-E15, PVP/VA and PVP K30 were identified as primary stabilizers while SLS, Soluplus, P407, P188, T20 and T80 were identified as secondary stabilizers. The concentrations of primary stabilizers (Y_1_) and the ratio of seconder stabilizer to primary stabilizer (Y_2_) were considered critical material attributes (CMAs). The factors with their low and high levels are presented in Table 1. A total of 42 experimental runs were conducted with a one-factor-at-a-time (OFAT) approach for screening and selection of stabilizers (Table 2).

#### 2.3.1. Wettability of CFZ by Stabilizer Solutions

Wettability is the capability of a liquid to spread across the surface of a solid and can be expressed as a contact angle [27]. Contact angle measurements were performed according to the methods of Yue et al. and Pardeike and Müller [28,29]. Briefly, 400 mg coarse CFZ was compressed to a disc with a single punch tablet machine. A droplet of water, stabilizer solutions and the combinations of stabilizer solutions were put on the surface of the disk and their images were captured by the optical tensiometer, Attention Theta Lite (Biolin Scientific, Espoo, Finland). The images are automatically analyzed by the sessile drop method and calculated as contact angle through Young’s equation. The measurements were performed in triplicate.

#### 2.3.2. Saturation Solubility Measurements

Each aqueous solution of stabilizers was prepared as mentioned in Table 2. The apparent saturation solubility of CFZ in these aqueous solutions was determined by the shake flask method [30,31]. An excess amount of coarse CFZ material in the vials was subjected to each stabilizer solution. Mixtures in the vials were shaken in a mechanical water bath shaker (WNB-7, Memmert GmbH, Schwbach, Germany) at 24 ± 1 °C and 100 rpm for a period of 24 h. Then, they were centrifuged at 16.000 rpm for 30 min using a high-speed tabletop centrifuge, Heraeus Biofuge Stratos (Thermo Fisher Scientific, Waltham, MA, USA). A certain amount of filtered supernatant was diluted with mobile phase and the drug concentration was analyzed by a validated assay method. The experiments were conducted in triplicate.

#### 2.3.3. Dynamic Viscosity Measurements

Viscosities of all stabilizer solutions (from N1 to N42) were measured using Brookfield viscometer (Model: DV2T, AMETEK Brookfield, Middleborough, MA, USA). The measurements were performed with LV-1 spindle in triplicate at 22 °C ± 2 °C [32].

### 2.4. Statistical Data Analysis

The experimental runs were created and all data were analyzed using Minitab Statistical Software (Version 19).

### 2.5. Characterization of Optimized CFZ Nanosuspension

#### 2.5.1. Particle Size, PDI and zeta Potential Measurements

The Z average particle size, PDI and zeta potential of the optimum CFZ nanosuspension were measured using Litesizer 500, as described in Section 2.3.

#### 2.5.2. Drug Content and Solubility of CFZ in Optimized Nanosuspension

The concentration of the optimum nanosuspension was analyzed as described in Section 2.4. The aqueous solubility of pure CFZ in both water and buffer solutions of pH 1.2, 4.5, 6.8 and 7.2 were performed.

#### 2.5.3. In Vitro Dissolution Test Study

The dissolution test was performed by modifying the method described in the Food and Drug Administration (FDA) database [33]. The study was conducted in 600 mL dissolution media at 37 ± 0.5 °C for 60 min under non-sink conditions using USP Apparatus II, Varian VK 7010 (Agilent Technologies, Santa Clara, CA, USA). Buffer solutions of pH 4.5 and pH 6.8 were preferred as non-sink dissolution media. An amount of 5 mL of aliquot was withdrawn at 1, 5, 10, 15, 20, 30, 45 and 60 of the time points and filtered using a 0.2 µm RC syringe filter (Sartorius, AG, Goettingen, Germany) (for example, [34,35]). The amount of dissolved CFZ was determined by a validated dissolution method in which the HPLC equipment conditions were kept the same as in Section 2.4 but the standard stock solution was prepared by mixing a small volume of methanol with each buffer solution and the standard solutions were prepared by diluting with each buffer solution.

#### 2.5.4. Differential Scanning Calorimetry (DSC) Analysis

Thermal properties of pure API, physical mixture (PM) and the optimized nanosuspension were characterized with DSC analysis using DSC1 instrument (Mettler-Toledo International Inc., Greifensee, Switzerland). To extract excess adsorbed stabilizers from the crystal surface, nanosuspension was centrifuged at 16.000 rpm for 30 min at 22 °C and washed 5 times with DI water after each time the supernatant was discharged (Heraeus Biofuge Stratos, Thermo Fisher Scientific, Waltham, MA, USA). The remaining part at the bottom of the vial was kept at room temperature overnight [36]. The sample weighing approximately 2 mg was placed in the aluminum pan, sealed and then subjected to heat in the range of 25 °C–200 °C at a heating rate of 10 °C/min under nitrogen purge gas flow (mL/min). An empty crimped pan was used as a reference.

#### 2.5.5. X-ray Powder Diffraction (XRPD) Analysis

Crystallinity properties of optimized nanosuspension were examined with a diffractometer (Rigaku Ultima IV, Rigaku Corporation, Tokyo, Japan). XRD patterns of CFZ API, PM and optimized nanosuspension were taken at a scan rate of 5 °C/min in the range of 0–50 °C.

#### 2.5.6. Stability Studies

The optimized nanosuspension was monitored at room temperature (22 °C ± 2 °C, 60% RH) and in a refrigerator (4 °C ± 2 °C) to evaluate physical stability for 3 months. Z average particle size, PDI and zeta potential were measured after one and three months [37]. All samples were analyzed in triplicate.

## 3. Results and Discussion

### 3.1. Preformulation Studies

Within the scope of preformulation studies, HPMC and SDS were the selected stabilizer agents as mentioned in the Introduction section. In detail, it was noted that HPMC and SDS were successfully used to obtain physically stable nanosuspension with an appropriate particle size [38,39].

HPMC is classified as generally regarded as safe (GRAS) by the FDA and its commercial types are identified by some codes regarding their grade of substitutions. E classes (hypromellose 2910) are the grade that have the highest ratios of methoxyl and hydroxypropyl, at 28–30% and 7–12%, respectively [40]. HPMC has the ability to form hydrogen bonding with the drugs through its methoxy- or hydroxypropyl groups. HPMC as a steric stabilizer forms a hydrodynamic boundary layer by adhering to the nanocrystal surfaces to protect the particles against aggregation [41]. SLS as an ionic surfactant shows an electrostatic repulsion effect between two drug particles and possibly acts as an absorption enhancer by opening tight junctions [39]. Moreover, managing SLS concentration is crucial for stable nanocrystals. SLS becomes a solubilizing agent by forming micelles if the concentration exceeds the critical micelle concentration (CMC) [42], and this could trigger the Oswald ripening.

After one hour of grinding in circulation mode, a thermodynamically favored nanosuspension was obtained with 1.25% HPMC E15 and 0.1% SLS. Z average particle size, PDI and mean zeta potential were 211.7 ± 5.73 nm, 0.141 ± 0.03 and −16.6 ± 0.75 mV, respectively. The mean particle size of the initial suspension was 28.412 µm, and after only one cycle, it dropped to 731.6 nm with a high effective agitator shaft speed, and the particle size reduction was monitored during the milling process, as presented in Figure S1a. In the presence of SLS and HPMC, the grinding process yielded acceptable nanosuspensions in terms of drug content, which was 96.50%.

The zeta potential of approximately ±20 mV is preferable to stabilize nanosuspension with steric and electrostatic stabilizers [43]. Although this formulation has a zeta potential of −16.6 mV, it was observed to have adequate stability along the short-term stability as shown in Figure S1. For instance, Karakucuk et al. also found a similar observation that the nanosuspension showed sufficient stability with a zeta potential below −20 mV [44]. The combination of HPMC and SLS exerts a powerful effect upon the negative value of zeta potential. SLS, an anionic surfactant, is charged negatively on the surface of drug particles and HPMC as a nonionic polymer, boosts the diffuse double layer thickness and hence decreases the zeta potential [18].

### 3.2. Selection of Final Stabilizers by Statistical Design of Experiment (DoE)

After obtaining a stable nanosuspension with a Z average particle size of 211.7 nm using 1.25% HPMC E15 and 0.1% SLS, the wettability of CFZ via the combination solution of HPMC and SLS, the saturation solubility of CFZ in the solution and the viscosity of the combination solution were measured and identified as target values for QbD approach. For example, Verma also showed in the study stabilizer type is a crucial parameter on the particle size and its impact relies on the parameters mentioned above [32].

HPMC, PVP/VA and PVP K30, as viscosity agents and SLS, Soluplus, T20, T80, P407 and P188 were selected as surfactants to much better stabilize the nanosuspension system together through a synergistic effect.

#### 3.2.1. Wettability of CFZ by Stabilizer Solutions

**Figure 1:**
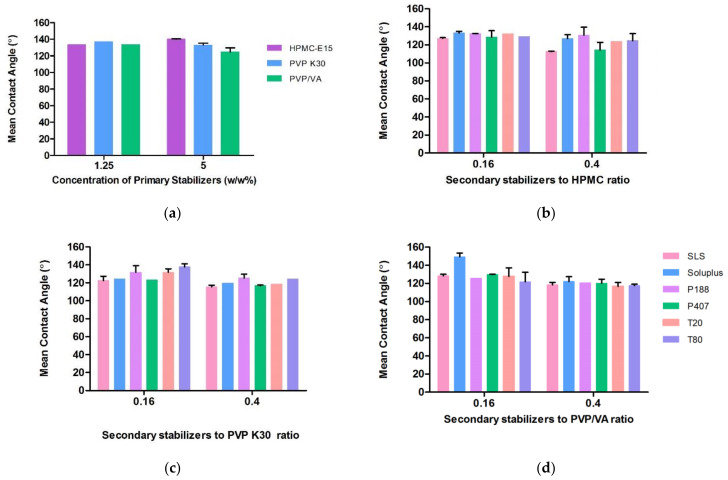
Measurements of contact angle: (**a**) CA measurements between drug and primary stabilizers; (**b**–**d**) CA measurements between drug and the combination of the surfactants with HPMC, PVP K30 and PVP/VA, respectively.

A minor contact angle (CA) generally means favorable wettability, and thus, the system can be easily stabilized [45]. The CA between a CFZ compact disk and water was measured as a control. All measurements with the solutions of polymers and surfactants show a lower contact angle than deionized (DI) water. The CA measurements are presented in Figure 1. CFZ exhibited poor wettability characteristics in water evaluating a CA range of 0°–180°, where 0° indicates entirely wettable and 180° entirely unwettable. Among the primary stabilizers at low concentrations, HPMC showed the lowest CA, while PVP showed the lowest CA when the concentration was increased to 5%. The increase in HPMC concentration resulted in an increased CA. Unlike PVP K30 and PVP/VA, an increasing concentration leads to a highly viscous HPMC solution; thus, it had difficulty diffusing onto CFZ particles. An increase in the primary polymer concentration led to a decrease in CA and the smallest CA was observed with PVP/VA.

**Figure 2:**
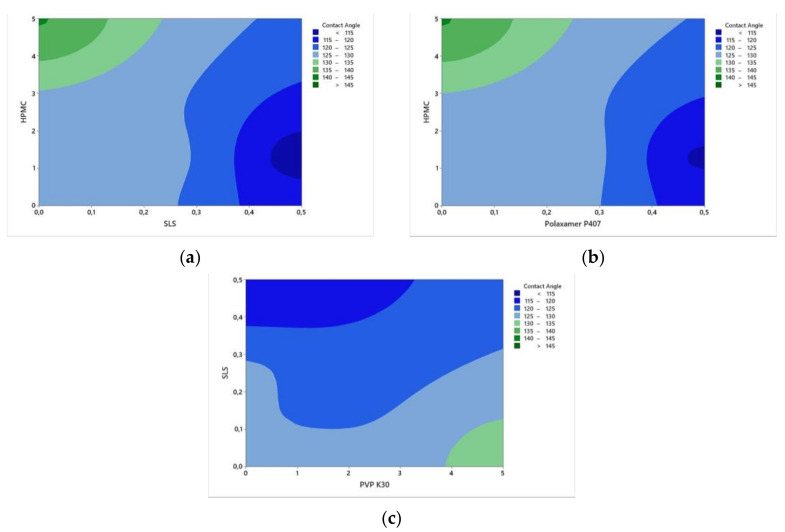
Contour plots of stabilizers on contact angle: (**a**–**c**) Effects of the combination of HPMC -SLS, HPMC-P407 and PVP K30-SLS on the contact angle, respectively.

**Figure 3:**
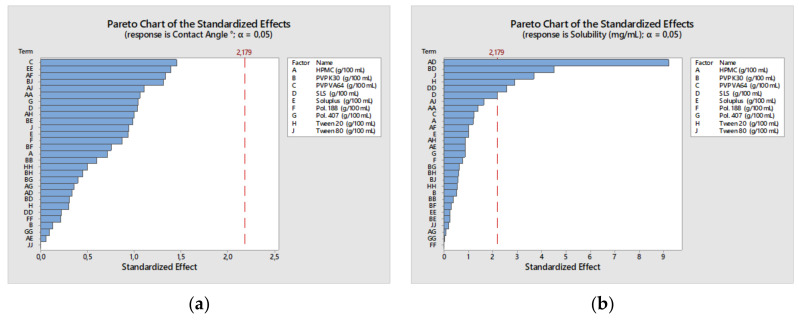

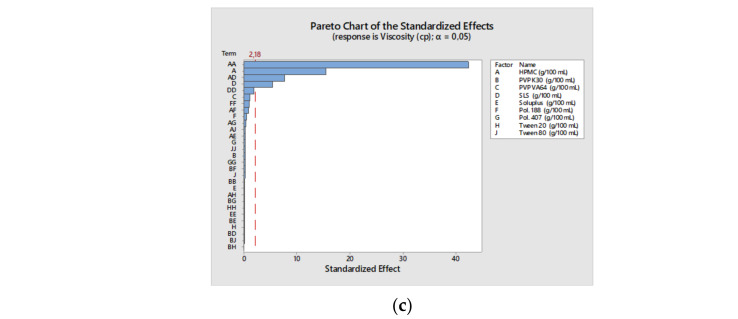
Pareto charts of CQAs: (**a**–**c**) Effects of the factors on the contact angle, saturation solubility and viscosity, respectively.

On the other hand, among the combination of stabilizer solutions, the CAs of CFZ were in the order of SLS: HPMC < P407: HPMC < SLS: PVP K30. The effects of SLS and P407 on wettability were similar as shown in the contour plots (Figure 2). It was expected that polymers and surfactants have a significant effect in obtaining smaller CA, but the Pareto chart showed that they affected CA non-significantly (Figure 3). This may be due to the greatly hydrophobic nature of CFZ. When considering CA between the water drop and CFZ compact disk (CA: 152.343°), even the aqueous stabilizer solutions with surfactants could not significantly decrease CA.

As there is no significant difference in CA results between solutions of the different types of stabilizers, it is likey that a higher concentration of stabilizer solutions can be used. But, higher concentrations of stabilizers were not preferred to avoid an excessive solubility of CFZ. Stabilizers, especially surfactants, offer a higher solubility for poor water-soluble drugs as well as particle wetting [46]. Both polymers and surfactants increased the solubility of CFZ as shown in the saturation solubility results. Therefore, this may be crucial for the instability of nanosuspension due to crystal growth or agglomeration following Ostwald ripening [47].

#### 3.2.2. Saturation Solubility

**Figure 4:**
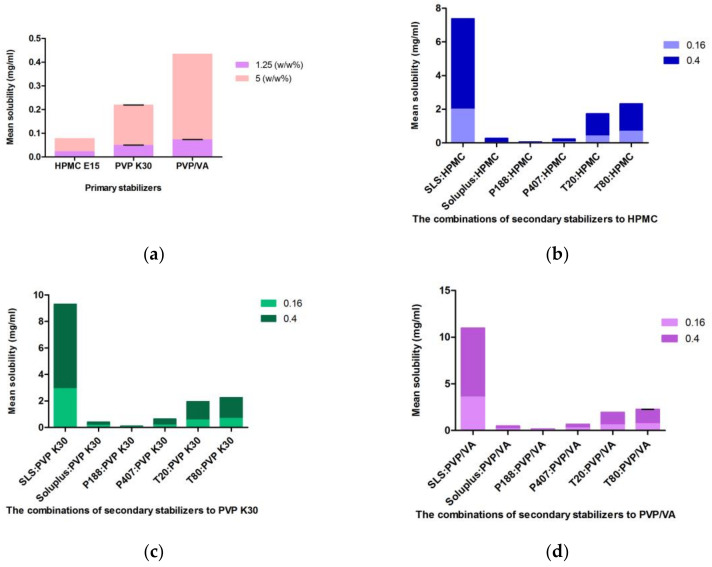
Measurements of saturation solubility: (**a**) Saturation solubility of the CFZ in primary stabilizers; (**b**–**d**), saturation solubility of the CFZ in the combination of surfactants and HPMC, PVP K30 and PVP/VA, respectively.

In the presence of polymers and surfactants, CFZ solubility was increased compared to the equilibrium solubility of CFZ in DI water (13.6 ± 0.3 µg/mL). An increase in polymer concentration from 1.25% to 5% led to a greater increase in CFZ solubility. But this effect was slightly lower in the HPMC solution as it has a higher viscosity than PVP K30 and PVP/VA solution. The saturation solubilities of CFZ with primary stabilizers were in the order of PVP/VA > PVP K30 > HPMC, while among the combination of stabilizer solutions, the highest solubility of CFZ was in PVP/VA and SLS, as presented in Figure 4. The two-way interaction showed that the combination of SLS/HPMC and SLS/PVP 30 significantly affected solubility, respectively (Figure 4). This was due to the efficacy of SLS as a solubilizer enhancer by forming micelles at interfaces with its hydrophobic tail and hydrophilic head groups. However, it should be taken into account that a higher solubility is not preferable for nanosuspension stability due to the higher rate of Ostwald ripening [48].

#### 3.2.3. Dynamic Viscosity

One of the critical factors that affect the grinding process is the viscosity of initial suspension, which depends on the solid volume fraction of drug particles and polymer concentration. In preliminary screening studies, drug concentration was kept at 5% to prevent the clogging of the grinding equipment. Moreover, it was observed that the grinding process was carried out with the powerful agitator shaft at an increasing HPMC concentration, but the product could not be recirculated. This was because the viscous product could not pass through the WAB separation system, thus resulting in clogging. Although viscous dampening can take place in any wet media mills, low-energy or planetary ball mills are especially susceptible to this case. Even after a much longer milling time, the drug nanocrystals may not be manufactured with high-viscosity grinding media [49]. Therefore, viscosity should be at a sufficient level to prevent the sedimentation of nanosuspension, but not reduce the grinding efficiency.

Mostly, the grinding yield increases as the viscosity increases [17]. But if the viscosity becomes too high, the energies supplied by grinding beads will not be adequate to launch breakage [50]. At larger viscosities, a major component of the kinetic energy of the grinding media is decreased by the movement of the suspension. Generally, grinding the drug particle to nanometers means that the stress intensity is sufficient to reduce particle size or disperse the agglomeration [51].

**Figure 5:**
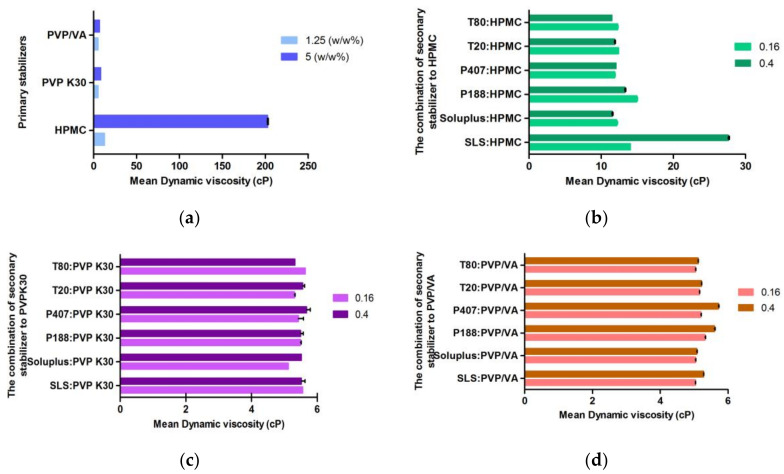
Measurements of dynamic viscosity: (**a**) Dynamic viscosity of the primary stabilizers; (**b**–**d**) dynamic viscosity of the combination of surfactants and HPMC, PVP K30 and PVP/VA, respectively.

The increase in HPMC concentration considerably increased the viscosity due to its cellulosic nature. Each aqueous combination solution of PVP/VA and PVP K30 showed a similar viscosity in the range of 4.6–5.8 cP, while the solution containing HPMC has a higher viscosity range of 10–20 cP, as shown in Figure 5. The viscosity of the obtained nanosuspension from the preformulations was measured and identified as a reference value for evaluating remaining stabilizer agents. The two-way interaction from Pareto charts showed that the combination of HPMC/HPMC and SLS/HPMC significantly affected viscosity, respectively (Figure 4).

**Figure 6:**
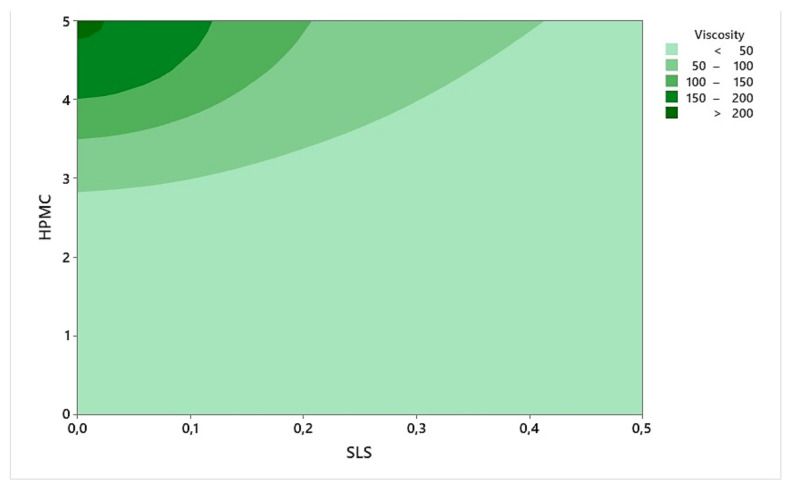
Contour plot of the effects of HPMC and SLS on viscosity.

The influence of SLS on viscosity was surprising, while the effectiveness of HPMC was normal due to its long-chain methoxyl and hydroxypropyl groups. Also, their interaction can be examined in the contour plot (Figure 6), and a dramatic increase in viscosity was observed from about 3% of HPMC concentration. And, it has an average viscosity of 15 cP with a concentration of 2%; hence, this allows us to obtain a nanosuspension in a suitable viscosity range at a low concentration.

The statistical software suggested an optimum formulation based on stable nanosuspension obtained with 1.25% HPMC and 0.1% SLS. The first optimum formulation comprised 0.857% HPMC, 3.947% PVP K30, 0.5% Soluplus and 0.439% P188 and was ground at the conditions mentioned in Section 2.2 for one hour. Unfortunately, visible degradation, aggregation and color changes were observed in the aliquots taken during milling (Figure S2). Considering the main component to be PVP K30 in the formulation, its hydroxyl and aldehyde groups at the terminal end may be responsible for the redox activity [52]. Also, PVP contains a small amount of peroxide as an impurity; therefore, generally, it can be rejected in the stage of formulation development according to the drug-excipient compatibility studies [53]. In this study, although compatibility studies between CFZ and PVP K30 and PVP/VA were performed, incompatibilities were not observed in the DSC studies since DSC is a restricted technique on its own for evaluating the interactions between API and excipients. A similar experimentation was also notified by Singhal et al. A stabilizer combination of SLS and PVP K30 was considered the first choice for nanogrinding, as the smallest CA was obtained by these stabilizers. But the drug-excipient compatibility study showed that there was a high degradation risk between PVP K30 and the related drug. In addition, it is well known that the risk of degradation is higher in liquid formulations such as nanosuspensions and also that a high energy input from grinding media will increase the rate of active ingredient degradation [17].

The optimization study was rebuilt after removing PVP K30 and PVP/VA. The second optimum formulation consisted of 1.299% HPMC, 0.022%SLS, 0.5% P407 and 0.082% T80. Compared to the first stable nanosuspension, it was observed that HPMC concentration was slightly increased, while SLS concentration decreased ~ 5 times, but T80 and P407 were included. Although it is not known how the algorithm works, these surfactants may help to better improve the wettability via a synergistic effect and keeping solubility at a certain level.

### 3.3. Characterization of Optimized CFZ Nanosuspension

The measured Z-average particle size after 60 min of grinding was 204.5 nm, the PDI value was 0.147, which is a marker of the narrow size distribution [54], and the zeta potential was −16.4 mV. 

**Figure 7:**
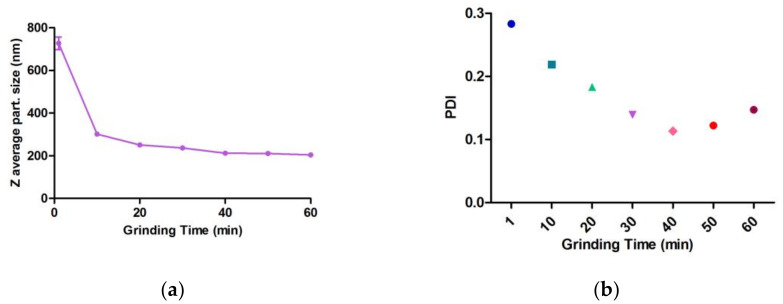
The pattern of CFZ grinding process versus time: (**a**) CFZ particle size versus time; (**b**) CFZ PDI versus time. The different color/shape dots show the time points corresponding to the x- axis during the milling process.

An alteration of CFZ particle size throughout grinding (Figure 7) showed a monotonous reduction, with the sharpest particle size reduction occurring in the first two cycles of the process, followed by a slow reduction in particle size up to 60 min of grinding. The particle size of the initial suspension was 30.949 µm, and after the first two milling cycles, it dropped to 727.5 nm and 301.5 nm, respectively. At first, the grinding process of CFZ was prompt as there is a large possibility of collision between CFZ particles and grinding beads, resulting in the formation of defects and fractures within the crystal construction.

After that, the grinding process decelerated, as the tiny crystals have a greater mechanical resistance and the grinding beads have less ability to capture these smaller particles [55]. The results showed that the combination of 1.299% HPMC, 0.022% SLS, 0.5%P407 and 0.082% T80 is good to maintain the stability of the nanosuspension. HPMC, as a steric stabilizer, showed excellent performance in covering the surfaces of nanocrystals. Considering that the main component of viscosity was HPMC, the viscosity of the nanosuspension was the best to rise the grinding efficiency and transfer the energy input from grinding media to drug particles. Also, P407 and T80 as non-ionic stabilizers contributed sterically to nanosuspension stabilization. P407 is an amphiphilic ABA-type block co-polymer containing poly (ethylene oxide) (A means PEO) and poly (propylene oxide) (B means PPO). Adsorption on the surface of the crystal is managed by hydrophobic PPO blocks, whereas hydrophilic PEO blocks enclosed the crystals supplying steric hindrance toward agglomeration/aggregation [56]. Although T80 presents less efficiency in stabilization due to a filmy adsorption layer, it combined well with other stabilizers at low concentrations [57].

The addition of a small amount of surfactants such as SLS, P407 and T80 is essential for the accomplished grinding process, owing to the extremely hydrophobic nature of CFZ. Also, considering the CA measurements in HPMC with secondary stabilizers at a low ratio, the smallest CA was in the rank of SLS < P407 < T80. The selection of these stabilizers by Minitab showed that they maximized the wettability of CFZ.

A few more trials were performed to gain more insight into the effectiveness of stabilizers or to test the Minitab’s ability to adjust the concentration of the secondary stabilizers (Table S1). API and HPMC concentration and process parameters were kept the same as in optimum nanosuspension. All additional trials showed a higher particle size than the calculated formulation by Minitab^®^, which probably indicates that this approach is more useful than applying the grinding process for selecting each stabilizer.

#### 3.3.1. Drug Content and Solubility of CFZ in Optimized Nanosuspension

The drug content of the optimized nanoformulation was 98.728%, which was a good result, since it was in the desired range (95–105%).

**Figure 8:**
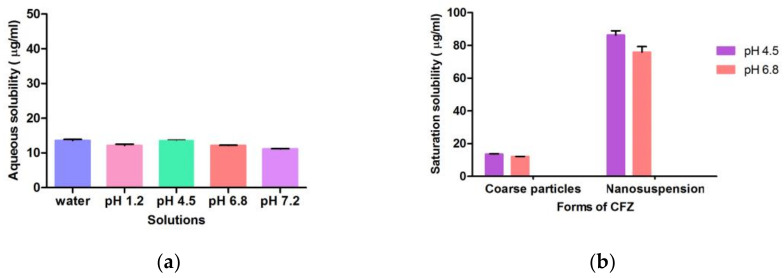
Solubility of CFZ: (**a**) Aqueous solubility of CFZ; (**b**) solubility of coarse CFZ and nanosuspension at both pH 4.5 and pH 6.8.

As presented in Figure 8a, CFZ is practically insoluble as its solubility is less than 15 µg/mL in water and at different pH values. Since CFZ reaches peak plasma concentration in 1–2 h, buffer solutions of pH 4.5 and pH 6.8 were considered as dissolution media. Comparing nanosuspension to coarse CFZ, it exhibited 6.34 and 6.22 times better solubility at pH 4.5 and pH 6.8, respectively (Figure 8b).

#### 3.3.2. In Vitro Dissolution Study

**Figure 9:**
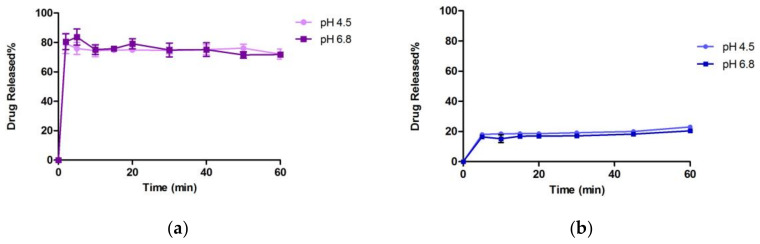

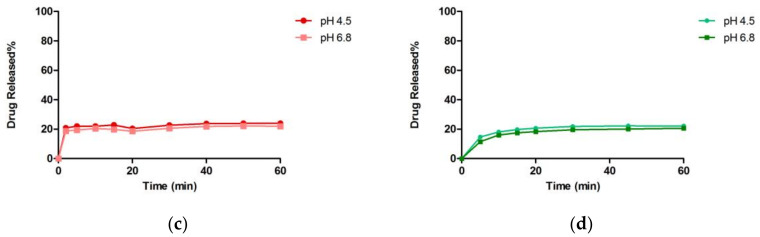
Dissolution profiles in non-sink conditions: (**a**) CFZ-NC; (**b**) marketed product; (**c**) microsuspension; (**d**) pure CFZ API.

Figure 9 presents the dissolution profiles of CFZ coarse drug, an optimized nanosuspension and marketed drug (Invokana^®^ 100 mg) in a buffer solution of pH 4.5 and pH 6.8. A total of 0.75% SLS is used as a sink condition in the dissolution method of CFZ. Such a large amount of SLS triggers the prompt dissolving of CFZ because of the micellar solubilization since 7.5 mg/mL SLS is far above its critical micellar concentration (CMC, 2.3 mg/mL). This rapid dissolution hides the difference in nanosuspension formulations. Hence, this dissolution method cannot discriminate in nanosuspension formulations with different particle sizes [9]. The non-sink conditions reduce the dissolution velocity and allow discrimination in dissolution profiles [58].

In a solution with pH 4.5, an optimized CFZ-NC presented a significant improvement in dissolution rate (79.318 ± 3.779%) in 60 min compared to the microsuspension (23.939 ± 0.213%) coarse CFZ (22.227 ± 0.297%) and marketed product (23.041 ± 0.644%). Similarly, in pH 6.8 buffer solution, an optimized CFZ-NC exhibited a considerable enhancement dissolution percentage (71.821 ± 1.263%) versus 22.080 ± 0.366%, 20.656 ± 0.850% and 20.483 ± 0.303%, respectively, in 60 min. Although coarse CFZ was extremely soluble in the combination of stabilizer solution, microsuspension (consisted of the stabilizer solution and coarse particles) showed very low dissolution rates at pH 4.5 and pH 6.8. Moreover, CFZ-NC exhibited 3.05 and 3.51 times better dissolution rates than the marketed drug product (Invokana^®^ 100 mg) in pH 4.5 and pH 6.8, respectively, although it showed a limited dissolution rate of the theoretical CFZ concentration due to non-sink conditions.

#### 3.3.3. Evaluation of the QbD Approach for Selecting Stabilizers in WSMM Process

The grinding chamber has a volume of 0.5 L and needs at least 0.6 L of initial suspension due to its cutdown in the chamber. CFZ is an expensive drug and testing each combination of the stabilizer solution is not feasible when considering large batches.

In pH 4.5 and pH 6.8 buffer solutions, an optimized CFZ-NC presented a significant improvement in dissolution rates (79.318 ± 3.779%, 71.821 ± 1.263%) in 60 min compared to the nanosuspension (prepared in preformulation study) with 1.25% HPMC-E15 and 0.5% SLS (42.825 ± 1.037%, 34.424 ± 0.010%), respectively. The dissolution profiles of nanosuspension with 1.25% HPMC-E15 and 0.5% SLS are given in the supplementary data (Figure S3). Although the first proposed combination of stabilizers was not compatible with CFZ in the wet-milling process, since we had no knowledge of their compatibility in the early development phase at high-stress energy, these dissolution results indicated that the QbD approach is still a material-saving, convenient and efficient roadmap for selecting stabilizer agents.

#### 3.3.4. DSC Studies

**Figure 10:**
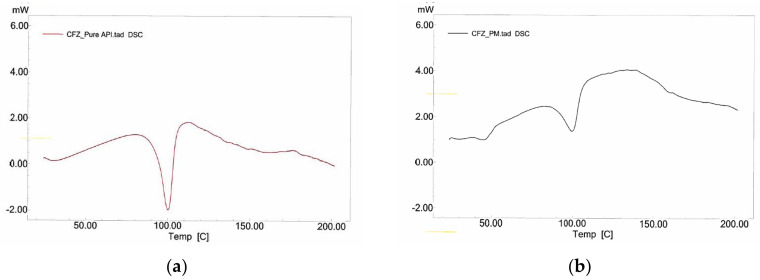

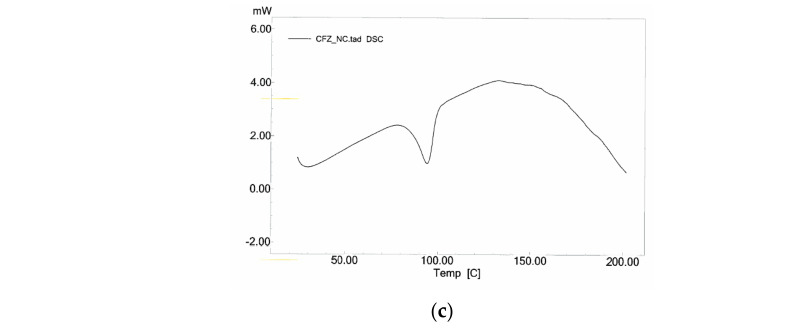
DSC thermograms of the materials: (**a**) pure API; (**b**) PM and (**c**) optimized nanosuspension.

DSC thermograms of pure API, physical mixture and dried nanosuspension are presented in Figure 10. A sharp endothermic peak between 105 and 107 °C was observed on the thermogram of pure CFZ, which confirms the melting point as mentioned in both the technical data package and the literature [59]. CFZ melting point as an indicator of its crystal structure was not only visible in the PM’s thermogram, but also in the CFZ nanosuspension.

#### 3.3.5. XRD Studies

**Figure 11:**
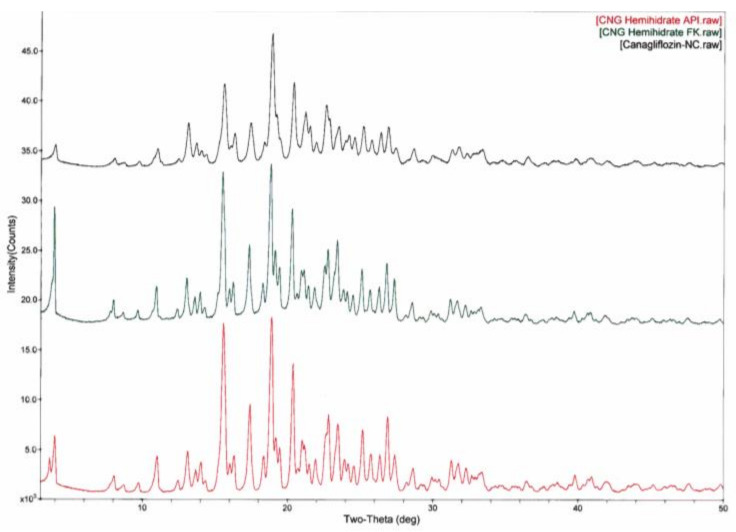
XRPD diffractograms of the materials: pure API, PM and optimized nanosuspension.

The XRD patterns of pure CFZ displayed distinct and sharp peaks for 2θ values at 15.98°, 19.05°, 19.39° and 21.25°, confirming its crystalline structure. Also, the characteristic peaks of pure CFZ were provided with the physical mixture of the optimum nanosuspension formulation. The diffractograms were compared to affirm the crystallinity upon PM and nanocrystals. In the nanocrystals, these characteristic peaks remained in the same position and were observed at low intensity as shown in Figure 11. When considering the DSC and XRD results, it was clearly observed that high-stress energy and pressure during the grinding process did not change the crystalline structure of API.

#### 3.3.6. Stability Studies

Table 3 shows the Z average particle size, PDI and zeta potential after 7 days, 14 days, one month and three months of storage of the optimum nanosuspension monitored at room temperature (22 °C ± 2 °C) and in the refrigerator (4 °C ± 2 °C).

Even after 3 months of storage, the particle size was acceptable both at room temperature and in the refrigerator, and the PDI value was below 0.25, which means it maintained a good particle size distribution. No aggregation was observed during both the grinding process and the storage period of 3 months, which may indicate that the concentration of secondary stabilizers was properly adjusted and SLS, T80 and P407 influenced each other synergistically because the physically stable nanosuspension with an appropriate particle size was obtained despite the low concentration of these stabilizers.

## 4. Conclusions

In this study, we established a systematic screening study for the selection of proper stabilizer agents using the QbD approach. The production of desirable nanosuspension with wet stirred media milling is a sophisticated phenomenon when considering process parameters and formulation components. Drug wettability, saturation solubility and dynamic viscosity as the factors related to the stabilizer agents were investigated using the OFAT approach and a Minitab optimization study was performed. The 204.5 nanosuspension with the uniform particle size distribution was obtained and the CFZ nanosuspension presented 3.05 and 3.51 times better dissolution rates in non-sink conditions than the marketed drug product (Invokana^®^ 100 mg). After grinding with high-stress energy and pressure, CFZ maintained its crystal structure and the optimized nanosuspension was stable for a period of 3 months at room temperature and in a refrigerator. Especially when the dissolution profiles of the nanosuspension with 1.25% HPMC and 0.5% SLS are compared with the optimized nanosuspension, it is clearly seen that using a few stabilizers at small amounts provide advantages in obtaining a stable nanosuspension with the smallest particle size. Also, the effects of the right stabilizer type and ratio on the particle size and dissolution rate were shown in this study. Therefore, all of these results may indicate that this screening study is useful and is a more feasible process than producing large batches with Dyno Mill^®^.

## Figures and Tables

**Table 1:** Independent factors and their levels for DoE.

Factors	Symbols	Range	
		Low (−1)	High (+1)
The concentration of primary Stabilizer (*w*/*w*%)	X_1_	1.25	5
Secondary/primary ratio	X_2_	0.16	4

**Table 2:** Experimental design with OFAT approach.

Experiment Name	Variables	The Concentration of Primary Stabilizers (*w*/*w*%)		Secondary/Primary Ratio	
		1.25	5	0.16	0.4
N1 */N2 **	HPMC E15	*	**	-	
N3 */N4 **	PVP K30	*	**	-	
N5 */N6 **	PVP/VA	*	**	-	
N7 */N8 *	SDS: HPMC	-		*	**
N9 */N10 **	Soluplus: HPMC	-		*	**
N11 */N12 **	P188: HPMC	-		*	**
N13 */N14 **	P407: HPMC	-		*	**
N15 */N16 **	T20: HPMC	-		*	**
N17 */N18 **	T80: HPMC	-		*	**
N19 */N20 **	SDS: PVP K30	-		*	**
N21 */N22*	Soluplus: PVP K30	-		*	**
N23 */N24 **	P188: PVP K30	-		*	**
N25 */N26 **	P407: PVP K30	-		*	**
N27 */N28 **	T20: PVP K30	-		*	**
N29 */N30 **	T80: PVP K30	-		*	**
N311 */N32 **	SDS: PVP/VA	-		*	**
N33 */N34 **	Soluplus: PVP/VA	-		*	**
N35 */N36 **	P188: PVP/VA	-		*	**
N37 */N38 **	P407: PVP/VA	-		*	**
N39 */N40 **	T20: PVP/VA	-		*	**
N41 */N42 **	T80: PVP/VA	-		*	**

* stands for low concentration and low ratio for primary stabilizers and secondary/primary stabilizers, respectively. ** stands for high concentration and high ratio for primary stabilizers and secondary/primary stabilizers, respectively.

**Table 3:** Z average particle size, PDI and zeta potential of CFZ nanosuspension after a one-hour milling of grinding measured freshly and after a period of 3 months (mean ± SD).

Sample Name	Particle Size (nm)	PDI	Zeta Potential (mV)
Fresh sample	204.6 ± 5.22	0.147 ± 0.50	−16.4 ± 0.1
After 7 days at 22 °C	226.1 ± 5.17	0.177 ± 0.26	−18.4 ± 0.2
After 7 days at 4 °C	231.6 ± 5.14	0.148 ± 0.40	−19.3 ± 0.0
After 14 days at 22 °C	235.8 ± 2.60	0.154 ± 0.21	−16.7 ± 0.1
After 14 days at 4 °C	225.8 ± 5.22	0.171 ± 0.49	−17.9 ± 0.2
After 1 month at 22 °C	248.7 ± 1.62	0.188 ± 0.26	−16.9 ± 0.2
After 1 month at 4 °C	241.4 ± 1.94	0.216 ± 0.13	−17.9 ± 0.3
After 3 months at 22 °C	261.3 ± 1.95	0.163 ± 0.29	−14.1 ± 0.2
After 3 months at 4 °C	261.5 ± 6.28	0.216 ± 0.35	−17.8 ± 0.3

## Data Availability

All data are contained within this article.

## Supplementary Materials

The following supporting information can be downloaded at: https://www.mdpi.com/article/10.3390/bioengineering10080927/s1, FigureS1: Measurements of the particle size and PDI of grinding nanosuspension; Table S1: The composition of additional trials and Z average particle size, PDI value; Figure S2: The degradation views of the first candidate optimum formulation; Figure S3: The dissolution profile of the nanosuspension developed in prefomulation study in non-sink conditions.
