# Supplementary material for: Systematic Screening Study for the Selection of Proper Stabilizers to Produce Physically Stable Canagliflozin Nanosuspension by Wet Milling Method
Source: Bioengineering (Basel). 2023 Aug 4;10(8):927. doi: 10.3390/bioengineering10080927 (PMC10451257; doi:10.3390/bioengineering10080927)
Supplement: Supplementary file 1 [file bioengineering-10-00927-s001.zip › bioengineering-2498976-supplementary.pdf]

bioengineering

MDPI

Article

Supplementary Data

Systematic Screening Study for the Selection of Proper Stabilizers to Produce Physically Stable Canagliflozin Nanosuspension by Wet Milling Method

Yagmur Pirincci Tok [1], Burcu Mesut [2], Sevgi Güngör [2], Ali Osman Sarıkaya [3], Emre Erol Aldeniz [3], Udaya Dude [3] and Yıldız Özsoy [2,*]

[1] Institute of Health Sciences, Istanbul University, Istanbul 34216, Türkiye; yagmur.pirincci@ogr.iu.edu.tr
[2] Department of Pharmaceutical Technology, Faculty of Pharmacy, Istanbul University, Istanbul 34126, Türkiye; bmesut@istanbul.edu.tr (B.M.); sgungor@istanbul.edu.tr (S.G.)
[3] Research and Development Center, Abdi İbrahim Pharmaceutical Company, İstanbul 34538, Türkiye; ali.sarikaya@abdiibrahim.com.tr (A.O.S.); emre.aldeniz@abdiibrahim.com.tr (E.E.A.); udaya.dude@abdiibrahim.com.tr (U.D.)
* Correspondence: yozsoy@istanbul.edu.tr; Tel.: +90-(212)-440-0272

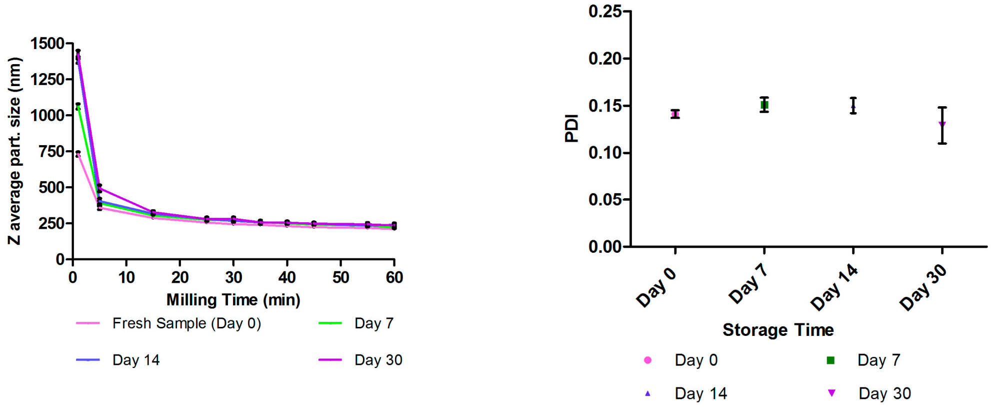

(**a**) (**b**)

Figure S1. Measurements of the particle size and PDI of grinding nanosuspension: **(a)** Particle size reduction profile versus time during milling and its stability over 1 month; **(b)** PDI values of final fresh nanosuspension and over 1 month

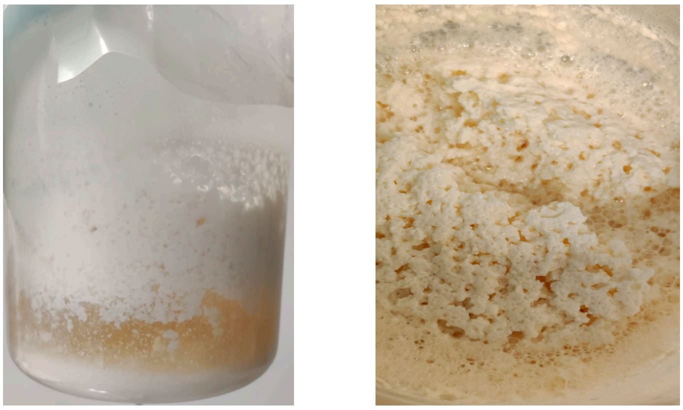

(**a**) (**b**)

Figure S2. The degradation views of the first candidate optimum formulation: **(a)** front view; **(b)** top view

Table S1. The composition of additional trials and Z average particle size, PDI value

Sample Run	SLS% (w/w)	T80% (w/w)	P407% (w/w)	Particle size (mean nm ± SD)	PDI (mean ± SD)
T1	0.075	0.01	0.2	230.2 ± 4.62	0.146 ± 0.13
T2	0.075	0.01	0.5	266.8 ± 11.37	0.196 ± 0.22
T3	0.05	0.025	0.5	216.5 ± 5.4	0.136 ± 0.44
T4	0.022	0.082	0.2	217.0 ± 3.36	0.118 0.43

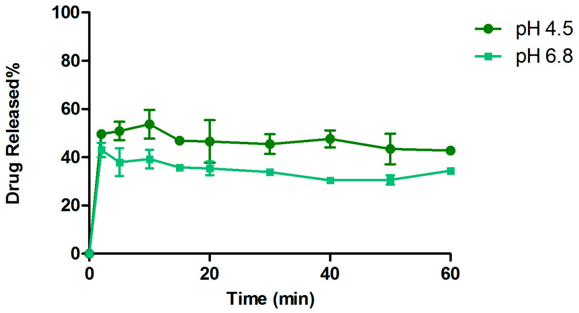

Figure S3. The dissolution profile of the nanosuspension developed in pre-fomulation study in non-sink conditions
